# Isolation of pathogenic bacteria from fomites in the operating rooms of a specialist hospital in Kano, North-western Nigeria

**Published:** 2012-07-28

**Authors:** Emmanuel Nwankwo

**Affiliations:** 1 Department of Microbiology and Parasitology, Faculty of Medicine, Bayero University, Kano, Nigeria

**Keywords:** Bacterial Pathogens, fomite, operating room, antibiotic susceptibility

## Abstract

**Background:**

Nosocomial infection constitute over 25% of infection rates in the hospital setting causing significant morbidity and mortality especially in developing countries. The aim of this study is to establish the possible presence of known bacteria pathogens on fomites in the operating theatre and evaluate their antibiotic susceptibility pattern.

**Methods:**

Various items in the operating theatre rooms such as forceps, scissors, floor, walls, suction tube, sink, theatre bed covers etc. were screened for the presence of bacterial and fungal pathogens from Murtala Mohammed Specialist Hospital, Kano between Jan – Aug 2009. One thousand eight hundred (1,800) samples were processed. Bacterial and fungal isolates were identified by standard microbiological procedures. Antibiotic susceptibility testing was carried out by disc diffusion method.

**Results:**

A total of eight bacteria genera and four fungal species were observed. The following bacterial pathogens were isolated; Escherichia coli (10.0%), Proteus Mirabilis (8.33%), *Proteus vulgaris* (6.70%), *Pseudomonas aeruginosa* (23.3%), *Staphylococcus aureus* (0.83%), Streptococcus spp. (18.3%), *E. faecalis* (3.33%), Coagulase negative staph (28.3%) and *Salmonella choleraesius* (0.83%). Ofloxacin and ceftriaxone showed encouraging results against the isolates.

**Conclusion:**

Fomites should be regarded as a possible source of nosocomial infection since bacteria from them can be carried from the hands of theatre personnel to the patient undergoing surgery or through redispersed bacteria from surfaces during surgery.

## Background

Inanimate objects which become contaminated with pathogenic bacteria and then spread infection to others are often referred to as fomites. Most outbreaks of infection associated with inanimate objects are caused by items that should be sterile but have been contaminated [1].

The hypothesis that environmental microorganism cause human diseases arises from two facts, firstly, our interaction with the inanimate environment is constant and close, secondly environmental objects are usually contaminated often with important human pathogens. Unfortunately, though it is fairly easy to assess the prevalence of microorganism in the environment, it is relatively difficult to establish the role the organisms in the environment play in causing human disease [2].

There are no systematic studies of the relative importance of various environmental factors in ensuring a safe operating room environment; however it is known that contaminated fluid or equipment in the operating room may result in contamination of surfaces and lead to outbreaks of wound infection with *Pseudomonas aeruginosa* or *Serratia marcescens* [3].

In the study carried out by Maki and his colleagues [4] in trying to assess the relationship between organism on environmental surface and nosocomial infection, they virtually ruled out the environment as a significant vector for nosocomial infections. However the limitations in the study is that the two pathogens for which environmental contact is of primary importance,*Aspergillus* and *Legionella* were not assessed neither were environmental culture processed for anaerobes e.g. *Clostridium difficille*, *Klebsiella* [5], *Pseudomonas* [6] and other gram negative organisms can be recovered from hospital areas, but the best correlation with patient acquisition is hand borne rather than air borne organism [7]. Although it has always been acknowledged that bacterial pathogens in fomites could be a possible risk factor in nosocomial infection, there is no study to confirm the presence of such pathogens in this locality. This is the focus of the present research.

## Methods

Sterile cotton wool swab sticks were prepared by making the cotton wool end wet with physiological saline. These were used to swab various items in the operating room theatre. From 30 sources in three (3) operating theatres, a total of 900 consecutive duplicate samples (1,800 samples) were collected. Sixty (60) samples were collected weekly before surgery for 30 weeks at the Murtala Mohammed Specialist Hospital MMSH, Kano between January and August 2009. While 1,620 samples were collected from 27 sources in the three operating rooms namely; Operating Lamp (OPL), Floor (FL), Wall (WL), Sink (SK), Suction tube (ST), Forceps (FC), Scissors (SC), Trolley (TR) and Anesthetic machine (ANM), one hundred and eighty (180) samples were obtained from three operation beds by sweep plate method. The theatre rooms screened were Main, Gynecology and Maternity. Two swab sticks were used for each item. While one was used for aerobic cultures, the other was used for fungal screening. Anaerobic cultures were not done. While the swab stick for bacterial culture were inoculated on MacConkey, Manitol salt and blood agar plates and incubated at 37°C for 18-24 hrs, those for fungal screening were inoculated on sabourad dextrose agar and incubated at room temperature for 72 hours. Samples for sweep plate method were collected with blood and sabourad dextrose agar plates. The former was incubated at 37°C for 18-24hrs while the later was incubated at room temperature for 72 hours.

Bacterial and fungal isolates were identified by standard microbiological techniques at Aminu Kano Teaching Hospital (AKTH) Kano [8]. Antibiotic susceptibility tests were carried out according to Bauer et al [9]. Overnight peptone water culture of the isolates were marched with McFarland turbidity standard 0.5 and spread over the surface of Mueller-Hinton agar with the help of a swab stick and allowed to dry. Antibiotic discs were placed on the surface of the medium with the aid of sterile forceps. This was incubated at 18-24 hrs at 37oC and the sensitivity plates interpreted by comparing the zones of inhibition according to CLSI 2010 Standards [10]. *Staphylococcus aureus* ATCC 25923, *Escherichia coli* ATCC 25922 and *Pseudomonas aeruginosa* ATCC 27853 obtained from AKTH were used as control organisms. Antibiotics (oxoid) disc potency were as follows Gentamicin(CN) 10µg, Ceftazidime(CAZ) 30µg, Ofloxacin(OFL) 10µg, Cloxacillin (CXC) 10µg, Ceftriaxone (CRO) 30µg, Ciprofloxacin (CIP) 10µg, Amoxycillin/Clavulanate (AMC) 30µg, Tetracycline (TE) 10µg, Cotrimoxazole (COT) 10µg, Cefuroxime(CXM) 30µg. Simple percentages were used to analyze the results.

## Results

Out of 900 samples screened for bacteria, 210(23.3%) yielded growth, while 120(57.1%) were recognized clinical pathogens. Also out of another 900 samples screened for fungal growth, 26(2.89%) yielded growth. A total of 1800 samples yielded 236(13.1%) fungal and bacterial isolates in the study. Table 1 shows the rate of contamination of various items in the Main theatre. The suction tube was the most contaminated with bacterial and fungal organisms, *Pseudomonas aeruginosa*, *Proteus mirabilis* and *Aspergillus* spp were observed. Table 2 shows the Gynaecology theatre with the least contamination of items. Both the forceps, scissors, trolley, anesthetic machine, operating lamp and walls showed no bacterial contamination. *Pseudomonas aeruginosa* and *Streptococcus* spp. were isolated from sink and suction tip respectively. Table 3 shows the items examined and the different bacterial and fungal isolates seen in the Maternity theatre. The suction tip was the most contaminated with bacterial infection such as *Enterococcus faecalis*, *Pseudomonas aeruginosa* and *Proteus vulgaris*. The forceps were not infected in the study. Table 4 shows microorganisms isolated from the theatre operation bed. The clinically important pathogens isolated from the theatre operating bed includes coagulase negative *Staphylococcus* (COANS), *Proteus* spp., *E. coli* and *Streptococcus* spp. The presence of fungi of various species, Rhizopus, Mucor and penicillin were also observed. Table 5 shows the pattern of bacterial pathogens isolated from fomites. Coagulase negative *Staphylococcus* was the most frequently isolated bacteria 34(28.3%) followed by *P. aeruginosa* 28(23.3%), *Strept* spp. 22(18.3%), *P. mirabilis* 12(10.0%) while the least were *S. aureus* 1(0.83%) and *Salmomella cholerasius* 1(0.83%). Table 6 shows the antibiotic sensitivity pattern of bacterial pathogens isolated from fomites in the operating room theatre. The quinolone (Ofloxacin) and the cephalosporin (Ceftriaxone) showed encouraging results. The tetracycline and cotrimoxazole show no sensitivity against the bacterial organism.

**Table 1:** Main theatre - frequency of recovery and distribution of microorganism isolated from formites in the operating rooms at Murtala Mohammed Specialist Hospital

	Frequency of occurrence (%) N = 30								
Organisms	OPL	FL	WL	SK	ST	FC	SC	TR	ANM
*Staphylococcus aureus*	0	0	0	0	0	0	0	0	0
COAN	0	0	0	0	0	0	3(100%)	0	0
*E. Faecalis*	0	0	0	0	0	0	0	0	0
*P. aeruginosa*	0	0	0	4(100%)	4(100%)	0	0	0	0
*S. choleraesius*	0	0	0	0	0	0	0	0	0
*Proteus Mirabilis*	0	0	0	0	2(16.6%)	0	0	0	0
*Proteus Vulgaris*	0	0	0	0	0	0	0	0	0
*Strept spp*.	4(100%)	0	4(100%)	0	0	0	0	0	0
*Penicillium*	0	2(13.3%)	0	0	0	0	0	2(28.6%)	0
*B. Circulans*	0	0	0	0	0	0	0	5(71.4%)	0
*Micrococcus*	0	8(53.3%)	0	0	0	0	0	0	0
*E. coli*	0	0	0	0	0	0	0	0	0
*Aspergillus spp*.	0	5(33.3%)	0	0	6(50.0%)	0	0	0	0
Total	4	15	4	4	12	0	3	7	0

OPL: Operation lamp; FL: Floor; WL: Wall; SK: Sink; ST: Suction tube; FC: Forceps; SC: Scissors; TR: Trolley; ANM: Anaesthetic machine

**Table 2:** Gyneacology theatre - frequency of recovery and distribution of microorganisms isolated from fomites in the operating rooms at Murtala Mohammed Specialist Hospital

	Frequency of occurrence (%) N = 30								
Organisms	OPL	FL	WL	SK	ST	FC	SC	TR	ANM
*Staphylococcus aureus*	0	0	0	0	0	0	0	0	0
COAN	0	0	0	0	0	0	0	0	0
*E. Faecalis*	0	0	0	0	0	0	0	0	0
*P. aeruginosa*	0	0	0	5(100%)	0	0	0	0	0
*S. choleraesius*	0	0	0	0	0	0	0	0	0
*Proteus Mirabilis*	0	0	0	0	0	0	0	0	0
*Proteus Vulgaris*	0	0	0	0	0	0	0	0	0
*Strept* spp.	0	0	0	0	2(100%)	0	0	0	0
*Penicillium*	0	0	0	0	0	0	0	0	0
*B. Circulans*	0	12(66.6%)	0	0	0	0	0	0	0
*Micrococcus*	0	6(33.3%)	0	0	0	0	0	0	0
*E. coli*	0	0	0	0	0	0	0	0	0
Total	0	10	0	5	2	0	0	0	0

OPL: Operation lamp; FL: Floor; WL: Wall; SK: Sink; ST: Suction tip; FC: Forceps; SC: Scissors; TR: Trolley; ANM: Anaesthetic machine

**Table 3:** Maternity theatre - Frequency of recovery and distribution of microorganisms isolated from fomites in the operation rooms at Murtala Mohammed Specialist Hospital

	Frequency of occurrence (%) N = 30								
Organisms	OPL	FL	WL	SK	ST	FC	SC	TR	ANM
*Staphylococcus aureus*	0	0	0	0	1(4.3%)	0	0	0	0
COAN	10(55.6%)	0	2(100%)	0	0	0	0	0	0
*E. Faecalis*	0	0	0	0	4(17.4%)	0	0	0	0
*P. aeruginosa*	0	0	0	5(71.4%)	10(43.5%)	0	0	0	0
*S. choleraesius*	0	1(4.7%)	0	0	0	0	0	0	0
*Proteus Mirabilis*	0	0	0	2(28.6%)	0	0	0	0	0
*Proteus Vulgaris*	0	0	0	0	8(34.8%)	0	0	0	0
*Strept* spp.	0	0	0	0	0	0	0	2(100%)	0
*Penicillium*	0	3(14.3%)	0	0	0	0	0	0	0
*B. Circulans*	8(26.6%)	15(71.4%)	0	0	0	0	0	0	0
*Micrococcus*	0	0	0	0	0	0	2(100%)	0	2(100%)
*E. coli*	0	2(9.5%)	0	0	0	0	0	0	0
Total	18	21	2	7	23	0	2	2	2

OPL: Operation lamp; FL: Floor; WL: Wall; SK: Sink; ST: Suction tip; FC: Forceps; SC: Scissors; TR: Trolley; ANM: Anaesthetic machine

**Table 4:** Microorganisms isolated from bedcover on the theatre operating room bed

	Frequency of occurrence (%)		
Organisms	Operation bed n=30		
	Main	Gyneacology	Maternity
*S. aureus*	0(0)	0(0)	0(0)
COANS	5(11.4)	4(36.4)	10(25.6)
*Bacillus* spp.	5(11.4)	0(0)	10(25.6)
*Microroccus*	20(45.5)	2(18.2)	4(10.2)
*Proteus* spp.	4(9.0)	0(0)	2(5.1)
*P. Putida*	0(0)	0(0)	2(5.1)
*Streptococcus* spp.	5(11.4)	0(0)	5(12.8)
*E. coli*	2(4.5)	4(36.4)	4(10.2)
*Rhizopus*	0(0)	0(0)	2(5.1)
Penicillium	3(6.8)	0(0)	0(0)
Mucor	0(0)	1(9.0)	0(0)
Total	44	11	39

DWM: D ward male; COANS: Coagulase negative staphylococci

**Table 5:** Pathogenic bacteria isolated from fomites in the operating room theatre at Murtala Mohammed Specialist Hospital

Bacteria Isolates	Number isolated	Percentage (%)
*COAN Staph*	34	28.3
*P. aeruginosa*	28	23.3
*P. mirabilis*	10	8.33
*Strept spp*.	22	18.3
*S. aureus*	1	0.83
*E. faecalis*	4	3.33
*S. cholerasius*	1	0.83
*P. vulgaris*	8	6.70
*E. coli*	12	10.0
Total	120	100

COANS: Coagulase negative staphylococci

**Table 6:** Antibiotic sensitivity pattern of bacteria pathogens isolated from fomites in the operating rooms theatre at Murtala Mohammed Specialist Hospital

Isolates	No. tested	Number and proportion of isolates sensitive to (%)									
		AMC	OFX	CXM	CLX	TET	CAZ	CN	CIP	CRO	COT
*E coli*	12	5(41.6)	9(75.0)	3(25.0)	NT	0(0)	6(50.0)	4(33.3)	5(41.6)	7(58.3)	0(0)
*Proteus mirabilis*	10	4(40.0)	7(70.0)	2(20.0)	NT	0(0)	7(70.0)	5(50.0)	6(60.0)	8(80.0)	0(0)
*Proteus vulgaris*	8	3(37.5)	5(62.5)	4(50.0)	NT	0(0)	5(62.5)	4(50.0)	6(75.0)	6(75.0)	0(0)
*S. aureus*	1	0(0)	1(100)	0(0)	0(0)	0(0)	0(0)	0(0)	1(100)	0(0)	0(0)
*P. aeruginosa*	28	0(0)	20(71.4)	0(0)	NT	NT	16(57.1)	10(35.7)	6(21.4)	18(64.2)	NT
*E. faecalis*	4	1(25.0)	2(50.0)	0(0)	1(25.0)	0(0)	2(50.0)	1(25.0)	2(50.0)	2(50.0)	0(0)
*COANS*	34	4(11.7)	30(88.2)	0(0)	8(23.5)	0(0)	16(47.0)	10(29.4)	16(47.0)	25(73.5)	0(0)
*S. cholerasius*	1	0(0)	1(100)	0(0)	NT	0(0)	0(0)	0(0)	0(0)	0(0)	0(0)
*Strept spp*.	22	15(68.1)	13(59.0)	12(54.5)	12(54.5)	0(0)	10(45.4)	8(36.3)	11(50.0)	8(36.3)	0(0)

AMC: Amoxycillin/clavulanate 30µg; OFX: Ofloxacin 10µg; CRO: Ceftriaxone 30µg; COT: Cotrimoxazole 25µg; CLX: Cloxacillin 10µg; CN: Gentamicin 10µg; CAZ: Ceftazidime 30µg; CXM: Cefuroxime 30µg; CIP: ciprofloxacin 10µg; TET: Tetracycline 25µg; COANS - Coagulase Negative Staphylococci; NT: Not tested

## Discussion

This study confirmed that various inanimate objects in the operating room theatre associated directly or indirectly with surgical procedures were variously contaminated with known bacterial and fungal pathogens. Although the direct involvement of these formites in disease transmission was not investigated in this study, the isolation of Coagulase Negative staphylococcus (COANS), *Pseudomonas aerugionosa*, *Proteus mirabilis*, *Strept* spp., S. aureus, E. feacalis, *Salmonella choleraesius*, *Proteus vulgaris* and *E. coli* including Aspergillus spp presents a serious concern for possible nosocomial transmission.

Some researchers [11] concluded in their study that the common nosocomial pathogens may well survive or persist on surfaces for months and can thereby be a continuous source of transmission if no regular prevention surface disinfection is performed. Although a researcher [12] remarked that the inanimate environment has little relevance to the spread of infection, other workers [13] noted that the fomites are involved in the transmission of pathogens in health care environments.

The results from this study in which established bacterial and fungal pathogens were isolated from fomites agrees with the findings of some workers [14] but varies with the reports of other researchers [15] who did not find any pathogenic bacteria on fomites from their study.

The presence of fungi as seen in the study confirms the contamination of air from outside the theatre. This same observation was made by some researchers [15]. In the present study, it was observed that the infection rate in the different operating theatre corresponded with the level of contamination of fomites observed. While the maternity ward had an infection rate of 38.4%, the main ward for general and specialist surgery had 33.8% followed by 17% in the gyneacology ward. Although these findings may not be as a direct result of contaminated fomites since the first two operating rooms receive a higher number of patients and more emergencies. Infection control practices should be strictly enforced. The incidence of multidrug resistance in gram negative bacilli and ESBL producers from clinical isolates is generating a lot of interest of late. Efforts should be made to ensure strict infection control practices whereby surfaces should be cleaned regularly to decontaminate fomites.

## Conclusion

The finding of established bacterial and fungal pathogens on fomites portends danger for surgical patients. These pathogens can easily acquire antibiotic resistance and constitute a threat to the life of patients if they eventually find their way as aetiologic agents of surgical site infection. It will be necessary to establish regular surface cleaning intervention as part of effective infection control policy

## References

[1] Barrie D (1994). Contamination of hospital linen by Bacillus cereus. Epidemiol Infect.

[2] Rhame FS, Beneth JV, Brachman PS (1992). The inanimate environment in Hospital infections. Little Brown and company (Inc).

[3] Ehrenkranz NJ (1980). Antibiotic sensitivity Serratia marcescens infection complicating cardopulmunary operation: contaminated disinfectant as a reservoir. Lancet.

[4] Maki DG (1982). Relation of the inanimate hospital environment to endemic nosocomial infections. N Engl J med.

[5] Turner AG, Craddock JG (1973). Klebsiella in a thoracic ICU. Hospitals.

[6] Dexter F (1971). Pseudomonas aeruginosa in a regional burn centre. J Hyg (Lond).

[7] Bauer TM, Ofner E, Just HM, Just H, Daschner FD (1990). An epidemiological study assessing the relative importance of airborne and direction contact transmission of microorganism in a medical intensive care unit. J Hosp Infect.

[8] Cheesbrough M (1993). Medical Laboratory Manual for tropical countries co- published by Tropical Health Technology. Microbiology..

[9] Bauer WA (1966). Antibiotic susceptibility by standard single disc method. Amer J Clin Path.

[10] CLSI – Clinical and Laboratory standards institute (2010). Performance standards for antimicrobial susceptibility teating. Twentieth informational supplement.

[11] Kramer A (2006). How long do nosocomial pathogens persist on inanimate surfaces? A systematic review. BMC infectious diseases.

[12] Ayeliffe GAJ (1991). Role of the Environment of the operating suite in surgical wound infection. Reviews of infectious diseases.

[13] Prescott LM, Harley JP, Klein DA (1999). Microbiology.

[14] Orji MU (2005). Isolation of pathogenic bacteria from hospital staff apparel in NIGERIA. Malawi medical journal.

[15] Njoku-Obi AN, Ojiegbe GC (1993). Bacterial air contamination of operating theatres and surgical wards of a University Teaching Hospital. Afr J Med Med Sci.

